# Paired mechanical and electrical acupuncture of neurogenic spots induces opioid-mediated suppression of hypertension in rats

**DOI:** 10.1186/s12576-020-00735-4

**Published:** 2020-02-06

**Authors:** Joo Hyun Shin, Yu Fan, Do-Hee Kim, Han Byeol Jang, Suchan Chang, Yeonhee Ryu, Jong Han Bae, Sanghag Lee, Bong Hyo Lee, Scott C. Steffensen, Chae Ha Yang, Hee Young Kim

**Affiliations:** 1 Department of Physiology, College of Korean Medicine, Daegu Haany University, Daegu, 42158 South Korea; 2 Clinical Medicine Division, Korea Institute of Oriental Medicine, Daejeon, 34054 South Korea; 3 Department of Physics, Yeungnam University, Gyeongsan, Gyeongbukdo 38541 South Korea; 4 TriBell Lab, Yeungnam University, Gyeongsan, Gyeongbukdo 38541 South Korea; 5 Department of Psychology and Neuroscience, Brigham Young University, Provo, UT 84602 USA

**Keywords:** Hypertension, Opioid system, Mechanical stimulation, Neurogenic spots, vlPGA, rVLM

## Abstract

While our recent studies have suggested that effective acupoints display neurogenic inflammation and can be identified as neurogenic spots (Neuro-Sps), the optimal stimulation conditions and the underlying mechanisms remain uncharacterized. We developed a combined mechano-electrical acupuncture device (MEA) and examined the effects of acupuncture at Neuro-Sps on systolic blood pressure (BP) in a rat model of immobilization-induced hypertension (IMH) and the mediation of endogenous opioid systems in its effect. Cutaneous neurogenic spots were found mostly in the forelimb. Electrical and mechanical acupuncture of Neuro-Sps increased 22-kHz ultrasonic vocalizations (USVs), c-Fos expression and cell excitability in the midbrain and synergistically alleviated the development of hypertension following immobilization stress, which was prevented by administration of the opioid antagonist naloxone into the rostral ventrolateral medulla (rVLM). These findings suggest that mechanical and electrical stimulation at Neuro-Sps suppresses the development of hypertension via mediation of the endogenous opioid system.

## Introduction

Acupuncture in oriental medicine traces back to several thousand years and has been practiced to treat a variety of conditions. Acupuncture stimulates certain skin areas called acupuncture points or acupoints [1, 2]. According to oriental medical theory, each acupoint communicates with a specific visceral organ; an acupoint reflects the status of a visceral organ, and visceral disorders can be treated by manipulating acupoints [1, 3, 4]. Although there have been considerable efforts to identify acupoints, the anatomical structures of acupoints are largely unknown. On the other hand, visceral disorders frequently produce a referred pain at topographically distinct somatic sites [5] due to the convergence of visceral and somatic afferents on the same neuron in the sensory pathway [6]. In multiple sites of skin overlying the referred pain, well-localized painful spots, known as neurogenic inflammation (neurogenic spots), are found and can be visualized experimentally in the skin by systemic injection of Evans blue dye (EBD) [7]. The neurogenic spots are characterized by plasma extravasation and vasodilation in the skin microvasculature and wheal-and-flare reaction arising from release of calcitonin gene-related peptide (CGRP) and substance P (SP) from activated sensory C-fiber terminals [8]. Our recent studies demonstrated that the neurogenic spots show hypersensitivity, high electrical conductance, and C-fiber-mediated sensations [9, 10]. Furthermore, when the needles inserted into neurogenic spots are stimulated manually or electrically, it generates therapeutic effects in a similar manner as acupoints [9, 11, 12]. Thus, we have proposed that the neurogenic spots function as acupoints [9, 10, 12].

In traditional acupuncture, needles have been manually or mechanically stimulated by the hand of the acupuncturists, called manual acupuncture (MA). Over the past several decades, manual acupuncture (MA) has been replaced with electroacupuncture (EA) in basic research and clinics, since EA has the advantages of high reproducible stimulus and low individual variations among physicians [13]. To solve some of the control issues of MA, we have developed a device that mimics manual MA via activation of mechanoreceptors whose signals are conveyed via large afferents in the ulnar nerve, which has been used previously to reduce cocaine-induced psychomotor responses in rodents [14, 15].

While our previous studies have shown that mechanical or electrical stimulation of neurogenic spots generates therapeutic effects in a similar manner as MA [9, 11], the optimal stimulation conditions for neurogenic spots, and the underlying mechanisms remain uncharacterized, which may be important for clinical application of visualized neurogenic spots for the treatment of various disorders. To investigate whether MA, EA, or combined MA + EA of Neuro-Sps might have therapeutic effects or synergic effects, we developed a new device (named mechano-electroacupuncture instrument; MEA) that enabled MA, EA or combined MA + EA. By using the MEA, we compared the effects of MA, EA or combined MA + EA at Neuro-Sps on systolic blood pressure in a rat model of immobilization-induced hypertension, and explored the mediation of endogenous opioid systems.

## Materials and methods

### Animals

Adult male Sprague–Dawley rats (Hyochang, Seoul, Korea) weighing 250–340 g were used. Animals were housed at constant humidity (40–60%) and temperature (22 ± 2 °C) with 12 h light/dark cycle and allowed free access to food and water. All experiments were carried out in accordance with the National Institutes of Health Guide for Care and Use of Laboratory Animals and approved by Institutional Animal Care and Use Committee (IACUC) at the Daegu Haany University.

### Chemicals

Evans blue dye (EBD; 50 mg/ml saline; Sigma-Aldrich, MO, USA); rabbit anti-c-Fos primary antibody (sc-52, Santa cruz, CA, USA); donkey anti-rabbit Alexa Fluor 594 (A21207, Life Technologies, CA, USA); naloxone (4 mg/ml saline, Sigma-Aldrich; a non-specific opioid receptor antagonist) were used in this study.

### Detection of neurogenic spots in the skin by EBD injection

Cutaneous Neuro-Sps were visualized by injecting Evans blue dye (EBD; 50 mg/kg, 50 mg/ml saline) as described previously [9]. While the rats were immobilized by the cone-shaped bags, the distal portion of the tail was dipped into 40 °C warm water for at least 30 s. EBD was then injected into the tail vein with a catheter (26 gauge), and skin color changes were observed up to 2 h after the injection. The blue-dyed spots on the skin were photographed and compared with an acupoint chart based on the transpositional method, which locates acupoints on the surface of animal skin corresponding to the anatomic site of human acupoints [16].

### Development of a novel mechano-electrical acupuncture instrument (MEA)

**Fig. 1:**
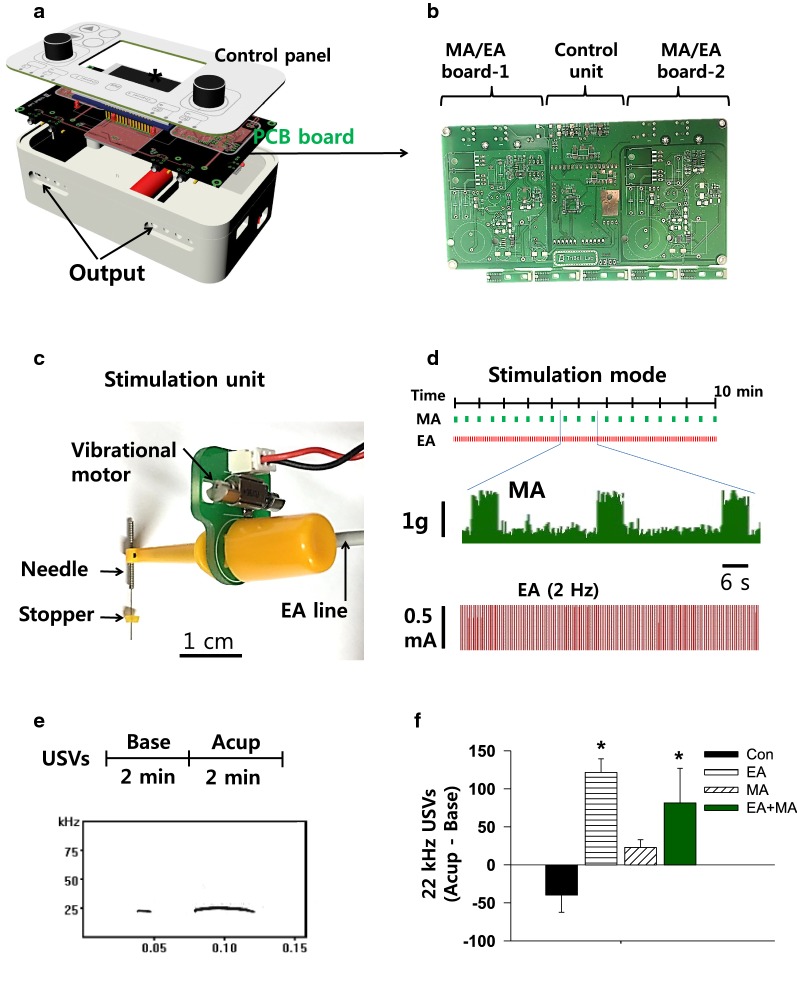
A mechano-electrical acupuncture (MEA) instrument **a** Three-dimensional images of the MEA. **b** Control board. Two pairs of mechanical and electrical acupuncture drive circuits were mounted on printed circuits boards. **c** Stimulation unit. A vibrator was combined with electrodes and a rubber grommet was fixed to the needle at a distance of 3 mm from the tip for controlling the depth of acupuncture needle insertion. **d** Stimulation mode and measurement of intensity or frequency during mechanical or electrical stimulation. In mechanical acupuncture (MA) mode, the needles were vibrated for 6 s every 30 s for a total of 10 min. For electrical acupuncture (EA) mode, electrical stimulation (2 Hz, 0.5 mA, 0.1 ms, triangular pulses) was applied to the needles for 10 min. Mechanical force (middle panel) and electrical pulse (lower panel) measured by a force transducer and an oscilloscope, respectively. **e**,** f** Measurement of 22-kHz ultrasonic vocalizations for each 2 min before (Base) and during acupuncture treatments (Acup). Representative 22-kHz USVs during acupuncture treatment (**e**). Base, baseline; Acup, acupuncture. The numbers of 22-kHz USVs during acupuncture treatments (**f**). The data were calculated by subtracting the number of basal USVs (before stimulation) from that of the USVs during acupuncture treatment. *p < 0.05 vs. Con; Con, handling only without acupuncture, *n* = 6; EA, electroacupuncture at neurogenic spots, *n* = 6; MA, mechanical acupuncture at neurogenic spots, *n* = 6; MA + EA, combined treatment of MA and EA, *n* = 6

An MEA device was developed to stimulate acupuncture needles electrically and/or mechanically. This device consisted of a program control unit and two stimulation units (Fig. 1a). In the control unit, 2 pairs of mechanical and electrical drive circuits were mounted on printed circuits boards (PCB; Fig. 1b), packaged with a 3-dimensional (3D) printed plastic cage and controlled by our custom-made program. In the stimulation unit, a vibrator (approximately 80 rotations/sec; MB-0412 V, Motor bank, Korea) was combined with electrodes and a rubber grommet was fixed to the needle at a distance of 3 mm from the tip for controlling the depth of needle insertion (0.10 mm in diameter, 10 mm in length of needle and 10 mm in length of handle; Dongbang Medical Co., Korea) (Fig. 1c).

For acupuncture treatment, the rat was restrained in a cone-shaped plastic bag and acupuncture needles were inserted into Neuro-Sps and stimulated with MA, EA or combined MA + EA. For EA stimulation, electrical stimulation (2 Hz, 0.5 mA, 0.1 ms, triangular pulses) was applied to the needles for 10 min. For MA treatment, the needles were vibrated for 6 s every 30 s for a total of 10 min. For combined MA + EA treatment, intermittent MA (6 s every 30 s) was given during continuous stimulation of EA for 10 min. Control group (Con) were lightly restrained in the same manner as the acupuncture treatment, but without needle insertion. Non-neurogenic spots group received combined MA + EA at the surrounding tissue 3–5 mm distant from the neurogenic spots.

### Measurement of intensities of mechanical or electrical stimulation

To measure the intensities of mechanical stimulation (vibration) in MA mode, the tip of acupuncture needle was attached to a force transducer (FT-100, iWorx/CB Sciences Inc., NH, USA) and the signals during vibration were fed into bridged amplifiers (ETH-200, CB Sciences Inc., Dover, NH, USA), filtered between 10 and 200 Hz and quantified using a LabChart & Scope program (AD Instruments). To determine the electrical frequencies and intensities generated in EA mode, the electrodes of MEA stimulation units were connected to bridged amplifiers (ETH-200, CB Sciences Inc., Dover, NH, USA) and recorded using a LabChart & Scope program (AD Instruments).

### Recordings of ultrasonic vocalizations (USVs)

Ultrasonic vocalizations (USVs) emitted by rats in response to acupuncture stimulation were recorded using customized sound-attenuating chambers as previously described [17]. The chamber consisted of two boxes to minimize exterior noise (inside box: 60 × 42 × 42 cm, outside box: 68 × 50 × 51 cm). The ultrasonic microphone was positioned at the center of the ceiling of the chambers and recorded with the Avisoft-RECORDER software (Avisoft Bioacoustics). For 22-kHz USVs, the signals were band-filtered between 18 and 32 kHz and analyzed using Avisoft-SASLab Pro (version 4.2, Avisoft Bioacoustics). Animals (*n* = 6) were habituated for at least 30 min in the chambers prior to experiments. After the USVs were recorded for 2 min as baseline (Base), the acupuncture needles were bilaterally inserted into the wrist area and stimulated for 2 min in EA, MA or combined EA + MA mode (Fig. 1e). All rats received 4 treatments (Con, EA, MA, or MA + EA) over 4 days in random order. Data were calculated by subtracting the basal USVs (Base) from the numbers of USVs emitted during 2-min of acupuncture stimulation (Fig. 1f).

### Immobilization-induced hypertension and measurement of blood pressure

Hypertension was induced by immobilization with a cone-shaped polyethylene bag, as described previously [18]. Systolic blood pressure (BP) was measured non-invasively with a tail cuff blood pressure monitor (Model 47, IITC Inc., CA, USA). Briefly, the rat was placed in a chamber kept at 27 °C, and an occluding cuff and a pneumatic pulse transducer were positioned on the base of the tail. A programmed electrosphygmomanometer (Narco Bio-Systems Inc., TX, USA) was inflated and deflated automatically, and the tail cuff signals from the transducer were automatically collected every 10 min using an IITC apparatus (Model 47, IITC Inc.). The mean of two readings was taken at each BP measurement.

### Immunohistochemistry of c-Fos in ventrolateral periaqueductal gray (vlPAG) or rostral ventrolateral medulla (rVLM)

After measurement of blood pressure, the brains were taken out, fixed in paraformaldehyde (PFA), cryo-protected, cryo-sectioned 30 μm thick and incubated in blocking solutions containing 0.3% Triton X-100, 5% normal goat serum in 0.1 M PBS at room temperature for 1 h. The sections were incubated with primary antibody for c-Fos (1:200) overnight at 4 °C, followed by an incubation of secondary antibody with donkey anti-rabbit Alexa Fluor 594. All sections were cover-slipped with a mounting medium (Vector laboratories, Burlingame, CA, USA) and imaged in vlPAG or rVLM under a 20 × objective using a confocal microscope (Zeiss Axioskop, Oberkochen, Germany).

### Microinjection of naloxone into the rVLM

The head of the rat was fixed on a stereotaxic frame in prone position. For microinjection into rVLM (stereotaxic coordinates: posterior, − 12.72 mm; lateral, + 2 mm; deep, − 10 mm), the nose was deflected ventrally so that the dorsal surface of medulla could be leveled horizontally. A 1.0 mm burr hole was made − 12.72 mm to the bregma and ± 2 mm to the midline, a 26 gauge needle connected to a Hamilton syringe was inserted − 10 mm deep into bilateral rVLM. Naloxone (10 nM) was infused at a constant rate of 0.1 μl/min (CMA 100, Microinjection pump; kdScientific, MA, USA). After termination of experiment, the brain stem was removed, fixed in PFA for 2 h and immersed in 30% sucrose overnight. The brains were cryo-sectioned 30 μm thick and stained with toluidine blue. Injection site was identified under a microscope.

### In vivo extracellular single-unit recordings of rVLM neurons

Single-unit discharges of rVLM neurons were recorded in anesthetized rats, as described previously [19] with slight modifications. In brief, 1 h after immobilization, rats (*n* = 14) were anesthetized with an intraperitoneal (i.p.) injection of urethane (1.5 g/kg). A carbon-filament glass microelectrode (0.4–1.2 MΩ, Carbostar-1, Kation Scientific, USA) was stereotaxically advanced to the rVLM (stereotaxic coordinates: posterior, − 11.96  to − 12.80 mm; lateral, + 1.9– + 2.4 mm; deep, 9.8–10.6 mm). Single-unit activity from the discharges was discriminated, recorded and analyzed via a CED 1401 Micro3 device and Spike2 software (Cambridge Electronic Design, UK). After recording stable baseline for at least 5 min, the rat received either saline (*n* = 7) or naloxone (*n* = 7; 1 mg/kg, i.p.) and recorded for 5 min. After 2 min of electrical acupuncture stimulation (EA; 2 Hz, 0.5 mA, 0.1 ms, triangular pulses), single-unit discharges were recorded for 5 min. Only EA treatment was carried out during single-unit recording of rVLM neurons, because electromagnetic noise generated during running of DC motor in MA interfered electrical signals of rVLM neurons.

### Data analysis

Statistical analysis was carried out using SigmaPlot 12.5 software. All data are presented as means ± standard error of the mean (S.E.M) and analyzed by one- or two-way repeated measurement analysis of variance (ANOVA) followed by the post-hoc Tukey test. *P* < 0.05 was considered statistically significant.

## Results

### A novel mechano-electrical acupuncture instrument (MEA)

A device was newly constructed for simultaneous MA and EA, as shown in Fig. 1a–c. Prior to in vivo acupuncture experiments, we calculated the mechanical force and electrical patterns at the tip of acupuncture needle in MA or EA mode. The MEA generated a force of approximately 1.2 g for 6 s every 30 s in MA mode and repetitive pulses of 2 Hz, triangular pulse, 1.5 ms width and 0.5 mA in EA mode, respectively (Fig. 1d). To evaluate whether mechanical stimulation (MA) applied to PC6 acupoints elicits more distress or pain than conventional EA in rats, the numbers of 22-kHz ultrasonic vocalizations during treatment were examined in each mode in normal rats (*n* = 6; Fig. 1e). While EA and MA + EA significantly increased 22-kHz USVs, compared to control rats (one-way repeated ANOVA; *F*_(3,15)_ = 5.795, *P* = 0.008; EA vs. Con, *P* = 0.007; EA + MA vs. Con, *P* = 0.046; Con, handling only, but without acupuncture), MA did not increase 22-kHz USVs, compared to control and EA, respectively (*n* = 6/group; Fig. 1f), indicating that MA did not elicit excessive pain compared to conventional electrical stimulation.

### Effects of stimulation of neurogenic spots on systolic blood pressure in a rat model of immobilization-induced hypertension

**Fig. 2:**
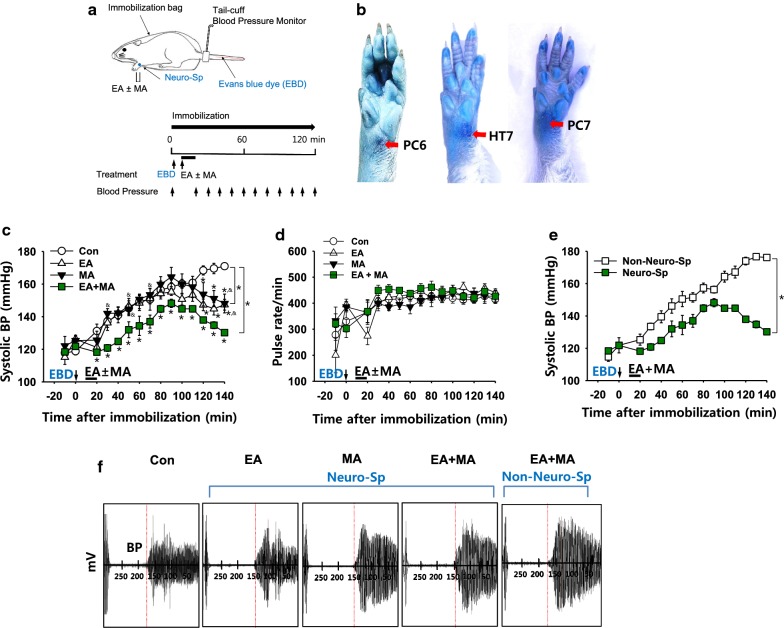
Effects of electrical and/or mechanical acupuncture at neurogenic spots on systolic blood pressure in IMH rats*. ***a** Schematic of the experimental procedure in the hypertension model. Evans blue dye (EBD) was injected via the tail vein after the initiation of restraint. Approximately 10 min after EBD injection, MA, EA or combined (MA + EA) was applied at Neuro-Sps on the forelimb for 10 min, and blood pressure was measured every 10 min. **b** Representative images of Neuro-Sps. Most blue dots were found in the forelimb, anatomically corresponding to classical acupoints including PC6, PC7 and HT7. **c** Effect of MA, EA, or combined MA + EA at Neuro-Sps on the development of hypertension in rats (EA, *n* = 8; MA, *n* = 8). The reduction of blood pressure was dominant in EA + MA group (*n* = 5). *p < 0.05 vs. Con. (immobilization only; n = 5); ^&^*P* < 0.05 vs. EA + MA. **d** Effect of MA, EA or combined MA + EA at neuro-Sps on the pulse rate in IMH rats. **e** Effect of MA + EA at neurogenic (*n* = 5) or non-neurogenic spots (*n* = 6) on the development of hypertension in rats (*p < 0.001 vs. Non-Neuro-Sp). The data of ‘Neuro-Sp’ are duplicated from ‘EA + MA’ of (**c**). **f** Representative pulse signals measured on the time points of 120 min after stimulation. *BP *blood pressure, *EBD* Evans blue dye

Cutaneous Neuro-Sps were detected by exploring the leakage of intravenously injected EBD after the initiation of immobilization (Fig. 2a). Neuro-Sps started to appear approximately 5 min after EBD injection, ranged in diameter from 0.5 to 3 mm and were maintained throughout the experiment in a rat model of immobilization-induced hypertension (*n* = 15), while these Neuro-Sps were not observed in normal rats (*n* = 6). When the Neuro-Sps were compared with the corresponding human anatomical acupoints, the most spots were found bilaterally or unilaterally on the wrist, and in acupoints of the forelimbs, such as PC6, PC7, and HT7 (Fig. 2b).

Next, we tested the effect of electrical and/or mechanical stimulation of needles inserted into Neuro-Sps on the development of systemic blood pressure (BP) in IMH rats. Immobilization stress in rats gradually increased systolic BP, reaching approximately 160 mmHg over the next 2 h (Con; Fig. 2c), consistent with our previous study [9]. When EA and/or MA were applied at Neuro-Sps near the wrist, it prevented or alleviated the development of hypertension, compared to control (Con; two-way repeated ANOVA; group *F*_(3, 12)_ = 4.719, *P* = 0.021; time *F*_(14, 56)_ = 47.102, *P* < 0.001; interaction *F*_(42, 168)_ = 3.186, *P* < 0.001; Fig. 2c), while no changes in pulse rates are observed following treatments (Fig. 2d). Moreover, intermittent MA during EA (MA + EA) at Neuro-Sps tended to show a synergistic effect on decreasing systolic BP, compared to EA or MA groups (two-way repeated ANOVA; group *F*_(2, 8)_ = 4.408, *P* = 0.051; time *F*_(14, 56)_ = 35.088,* P* < 0.001; interaction *F*_(28, 112)_ = 1.257, *P* = 0.2; Fig. 2c, f). However, MA + EA at non- Neuro-Sps 3–5 mm distal from Neuro-Sps over wrist failed to inhibit the development of hypertension, compared to the Neuro-Sp group (two-way repeated ANOVA; group *F*_(1, 4)_ = 38.002, *P* = 0.004; time *F*_(14, 56)_ = 62.503, *P* < 0.001; interaction *F*_(14, 56)_ = 19.737, *P* < 0.001; Fig. 2e, f).

### Effects of electroacupuncture, mechanoacupuncture or combined stimulation at neurogenic spots on c-Fos expression in ventrolateral periaqueductal gray (vlPAG) or rostral ventrolateral medulla (rVLM)

**Fig. 3:**
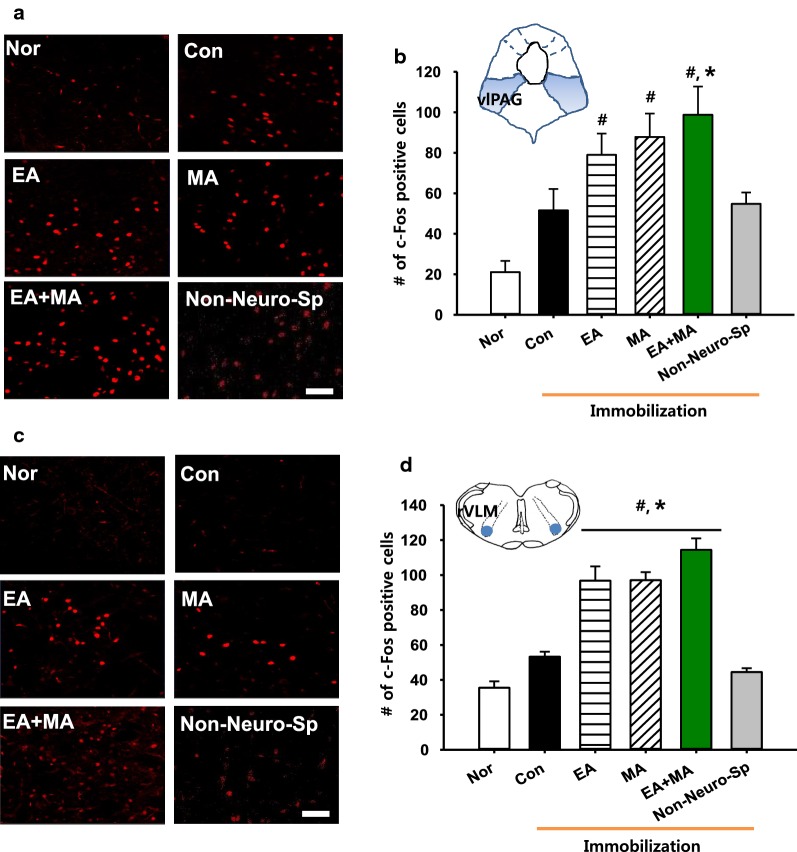
Effect of electrical, mechanical or combined stimulation at neurogenic spots on c-Fos expression in the vlPAG or rVLM in IMH rats*. ***a **c-Fos expression in the vlPAG following EA, MA or combined EA + MAMA at Neuro-Sps in IMH rats. **b** Quantification of c-Fos labeled neurons in vlPAG (shown in a) in each group. Data are expressed as the numbers of c-Fos-positive cells in vlPAG per mm^2^. **c** c-Fos expression in the rVLM following EA, MA or combined EA + MA at Neuro-Sps in IMH rats. **d** Quantification of c-Fos labeled neurons in rVLM in each group. Data are expressed as the numbers of c-Fos-positive cells in rVLM per mm^2^. *Nor* normal group without IMH (*n* = 7); *Con* control group, IMH only (*n* = 7); EA, electroacupuncture at Neuro-Sps in IMH rats (*n* = 8); *MA* mechanical acupuncture at Neuro-Sps in IMH rats (*n* = 7); EA + MA, electrical and mechanical acupuncture at Neuro-Sps in IMH rats (*n* = 7).#*P* < 0.05 vs. Nor; *P < 0.05 vs. Con. Bar = 50 μm

In another set of animals, to compare the extent of activation of the midbrain vlPAG the expression of c-Fos, a marker of neuronal activation [20], following MA, EA, and combined MA + EA was examined in the vlPAG of IMH rats (EA, *n* = 8; MA, *n* = 7; EA + MA, *n* = 7). The IMH rats tended to show a slight increase in c-Fos expression of vlPAG from that of normal rats (Nor, *n* = 7), although there was no significant difference between groups. MA, EA or combined MA + EA at Neuro-Sps increased c-Fos expression in the vlPAG, compared to controls (Con, immobilization only; one-way ANOVA, *F*_(3, 18)_ = 12.330, *P* < 0.001; Fig. 3a, b). In addition, MA + EA significantly enhanced c-Fos expression of vlPAG, compared to controls (one-way ANOVA, *F*_(1, 6)_ = 18.394, *P* = 0.005; Fig. 3a, b). c-Fos expression in the rVLM was also examined following MA, EA or combined MA + EA. Significant increases in the numbers of c-Fos positive cells were found in all acupuncture treatment groups (MA, EA and MA + EA, *n* = 5/Group), compared to normal (Nor) or control (Con, IMH) rats (one-way ANOVA, *F*_(5, 22)_ = 31.551, *P* < 0.001; Fig. 3c, d). This increase of c-Fos was dominant in the MA + EA group, while no significant increases in vlPAG or rVLM were observed in the rats given MA + EA at non-Neuro-Sps (*n* = 6; Fig. 3b, d).

### Role of endogenous opioids in rVLM in the inhibitory effects of neurogenic spot stimulation on elevated blood pressure

**Fig. 4:**
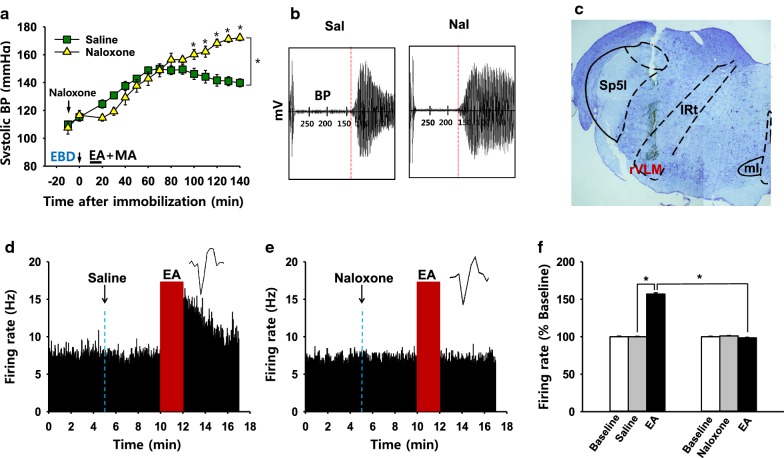
Effects of naloxone on anti-hypertensive effects by acupuncture or rVLM neuronal activity*.***a–c** Effects of intra-rVLM administration of naloxone on anti-hypertensive effects by EA + MA at Neuro-Sps**.** Representative pulse signals measured on the time points of 120 min after stimulation (**b**) and injection sites verified by toluidine blue stain (**c**). Either naloxone (*n* = 5) or saline (*n* = 5) was injected into rVLM 10 min before EA + MA treatment. *P < 0.05 vs. Saline. Although naloxone group slightly decrease blood pressure up to 40 min after naloxone administration, there are no differences in the blood pressure during the time points between naloxone and saline groups. **d–f** in vivo extracellular recordings of neurons in the rVLM. EA at Neuro-Sps increased the firing rate of rVLM neurons (*n* = 7, **d**,** f**), while pretreatment of naloxone prior to acupuncture prevented acupuncture-induced activation of rVLM neurons (*n* = 7, **e**, **f**). *P < 0.05

To determine whether anti-hypertensive effects of Neuro-Sp stimulation are mediated through endogenous opioids, we tested the effect of intra-rVLM naloxone, a non-specific opioid antagonist, prior to MA + EA treatment on systolic BP. While MA + EA treatment (Saline) applied to Neuro-Sps over wrist alleviated the development of systolic blood pressure in rats, intra-rVLM infusion of naloxone (Fig. 4c) blocked the inhibitory effects of acupuncture effects on hypertension (Naloxone; two-way repeated ANOVA; group *F*_(1, 4)_ = 3.747, *P* = 0.125; time *F*_(14, 56)_ = 74.756,* P* < 0.001; interaction *F*_(14, 56)_ = 13.776, *P* < 0.001; Fig. 4a, b). It suggests that the effects of acupuncture at Neuro-Sps on systolic BP are mediated via the endogenous opioid system of the rVLM in IMH rats.

Finally, to see whether acupuncture at Neuro-Sps excites rVLM neurons and that acupuncture effects might be mediated via endogenous opioids, we performed in vivo extracellular recording at rVLM and tested the effects of naloxone on rVLM excitability. When EA at Neuro-Sps near the wrist was applied for 2 min, single-unit discharges increased up to approximately 15 Hz and returned to baseline over 5 min after stimulation (Fig. 4d). On the other hand, acupuncture treatment 5 min after naloxone administration failed to increase the firing rate of rVLM, neurons compared to EA treatment after saline (*P* < 0.001; Fig. 4e, f).

## Discussion

The present study found that acupoints near the wrist, such as PC6, PC7 and HT7 displayed neurogenic inflammation in IMH rats. Electrical acupuncture or MA, or a combination of MA + EA at Neuro-Sps alleviated the development of hypertension in IMH rats. Moreover, combined MA + EA optimally reduced the elevated BP among treatment groups. Such stimulation activated vlPAG as well as rVLM neurons in midbrain. Moreover, the anti-hypertensive effects by stimulation of Neuro-Sps were prevented by intra-rVLM of naloxone. Naloxone also inhibited the enhanced excitability of rVLM induced by Neuro-Sp stimulation. Our findings suggest that paired electrical and mechanical acupuncture of Neuro-Sps effectively suppresses the development of hypertension in a rat model of IMH and such effects are mediated via endogenous opioids.

Consistent with our previous studies [10, 21], the present study showed that the majority of Neuro-Sps in hypertensive rats were found in the dermatome which is innervated by the same spinal segments (C8–T2) that innervate the heart [22] and those spots matched with acupoints, such as PC6, PC7, and HT7. These acupoints are prescribed most frequently for cardiac disorders [1] or proved to be effective in cardiovascular disorders [23, 24]. On the other hand, our previous studies showed that rats with colitis reveal Neuro-Sps mainly in hindpaw and frequently in the lower back, thighs or tail [9, 12] over the dermatome corresponding to spinal cord sections L2–S2, as mapped by electrical stimulation of C-fibers in spinal nerve in rats [25]. These results indicate that effective acupoints display neurogenic inflammation in the dermatome of segmentally related organs. Also, our recent study revealed that the increased conductance and temperature at the neurogenic inflammatory spots occur during the development of hypertension. The increase in conductance and plasma extravasation at acupoints in hypertensive rats was ablated by cutting median and ulnar nerves, blocking small diameter afferent fibers with resiniferatoxin injection into median and ulnar nerves, or antagonizing SP or CGRP receptors in acupoints [10]. Taken together, it suggests that acupoints over the wrist display active neurogenic inflammation through releasing neuropeptides SP and CGRP from small afferent fibers during the development of hypertension in IMH rats.

In our previous studies, when Neuro-Sps are stimulated electrically or manually, the increase in BP in the IMH rat or colonic inflammation in colitis rats is alleviated [9, 11]. The present study showed that simultaneous mechanical and electrical stimulation of Neuro-Sps produced synergic effects in IMH rats. There are several studies suggesting that the cardiovascular effects of acupuncture are due to activation of C-fibers. For example, the activation of C-fibers by injecting capsaicin, a transient receptor potential vanilloid 1 (TRPV1) agonist, or mustard oil, a TRP ankyrin 1 (TRPA1) agonist, into the skin with neurogenic inflammation over the median nerve blocks the development of hypertension in rats [9]. Similarly, electrical stimulation of PC5–6 acupoints near the wrist activate small afferent fibers in animal models to evoke cardiovascular effects [26] and the cardiovascular effects of EA are diminished in rats depleted of C-fibers by neonatal treatment with capsaicin [27]. In the present study, however, mechanical stimulation did not increase the numbers of 22-kHz USVs, an indicator of pain or discomfort in rodent [28]. It is not likely that synergic effects of mechanical and electrical stimulation on hypertension are due to more painful stimulation. We and others reported that transcutaneous electrical nerve stimulation (TENS) and electroacupuncture at PC6 activates A-delta (Aδ) and C-fibers to reduce hypertension [26, 29, 30]. Furthermore, our microneurographic study revealed that both A- and C-fibers of the median nerve are activated when TENS is applied to PC6 acupoint at low frequency. Furthermore, an experimental median nerve A-fiber block established prior to electrical stimuli at PC6 failed to prevent the BP-lowering effects of TENS. In turn, the application of capsaicin, a C-fiber activator, to the skin over the median nerve generates acupuncture-like effects on hypertension, suggesting a pivotal role of C-fibers in the reversal of hypertension [30]. Noxious mechanical stimulation is also able to activate C-fibers or the vibration-mechanical components (i.e., mechanical nociceptors in skin) [14]. Taken together, synergic effects of MA + EA on inhibition of systolic BP in IMH rats may be attributed to synergic activation of C-fibers by EA and MA.

While we showed the reduction of hypertension in a condition of electrical stimulation of forelimb (2 Hz, 0.1 ms), previous studies have suggested that when acupoints of hindlimb were electrically stimulated, the optimal frequencies of electrical stimulation were 5–10 Hz to evoke a depressor response of arterial pressure in pentobarbital-anesthetized rats [31]. In addition, Michikami et al. showed that increasing the pulse duration augments the reduction in arterial pressure and sympathetic nerve activity during electrical acupuncture at acupoints of hindlimb (i.e., Zusanli and Xiajuxu acupoints) [32] and suggested that pulse durations shorter than 2.5 ms did not change arterial pressure and sympathetic nerve activity, whereas durations over 2.5 ms decreased both the parameters immediately after the stimulation was started. This discrepancy in stimulation conditions for reduction of hypertension between our present and previous studies may be due to many vagaries including acupoint location (forelimb vs. hindlimb), treatment duration, stimulation intensity and use of anesthesia during stimulation.

The present study revealed that stimulation of Neuro-Sps near the wrist activated vlPAG and rVLM and injection of naloxone into rVLM reversed acupuncture effects on elevated BP. It is consistent with a previous study [33] demonstrating that EA at acupoints near the wrist induces c-Fos expression in the rVLM and PAG, especially in the vlPAG. They also showed that acupuncture stimulation increased c-Fos nuclei colocalized with rVLM perikarya containing enkephalin and in close apposition to fibers containing enkephalin or beta-endorphin in the rVLM and PAG. Furthermore, our in vivo extracellular recordings showing that stimulation at Neuro-Sps activated rVLM neurons in IMH rats, which were prevented by naloxone, suggest the involvement of endogenous opioids in neurogenic stimulation-induced changes of sympathoexcitatory cardiovascular reflexes in the rVLM. The rVLM plays an important role in regulating sympathetic outflow in the cardiovascular system [34]. The opioid peptides inhibit sympathetic outflow via the activation of μ-opioid receptors in the rVLM and decrease the sympathetic excitatory response induced by the activation of visceral afferents [35–37]. We and others have shown that stimulation of acupoints or Neuro-Sps near the wrist increases neuronal excitability in the rVLM [38]. In our previous study, when two different retrograde tracers were injected into Neuro-Sps on the wrist and apex of the heart in hypertensive rats, cardiac and somatic afferents from neurogenic spots converged on the same sensory neurons [9]. Premotor sympathetic cardiovascular neurons in the rVLM received convergent input from acupoints [38].

## Conclusion

In conclusion, Neuro-Sp stimulation reduces excitatory responses of those neurons to input from cardiac afferents through endogenous opioid and suppresses hypertension in IMH rats. The present study suggests that when Neuro-Sps are stimulated electrically and mechanically, it can generate therapeutic effects on hypertension via endogenous opioid system in a rat model of IMH.

## Data Availability

All data generated or analyzed during this study are included in this published article.

## Abbreviations

BP: Blood pressure
CGRP: calcitonin gene-related peptide
EA: Electroacupuncture
EBD: Evans blue dye
IMH: Immobilization-induced hypertension
MA: Manual acupuncture
MEA: Mechano-electrical acupuncture device
Neuro-Sps: Neurogenic spots
rVLM: Rostral ventrolateral medulla
SP: Substance P
USVs: Ultrasonic vocalizations
vlPAG: Ventrolateral periaqueductal gray
